# Left atrial function of patients with atrial fibrillation undergoing thoracoscopic hybrid ablation

**DOI:** 10.1093/icvts/ivae061

**Published:** 2024-04-03

**Authors:** Claudia A J van der Heijden, Bouke P Adriaans, Sander M J van Kuijk, Justin G L M Luermans, Sevasti-Marisevi Chaldoupi, Jos G Maessen, Elham Bidar, Bart Maesen

**Affiliations:** Department of Cardiothoracic Surgery, Maastricht University Medical Center, Maastricht, the Netherlands; Department of Radiology and Nuclear Medicine, Maastricht University Medical Center, Maastricht, the Netherlands; Department of Cardiology, Maastricht University Medical Center, Maastricht, the Netherlands; Cardiovascular Research Institute Maastricht, Maastricht, Netherlands; Department of Clinical Epidemiology and Medical Technology Assessment, Maastricht University Medical Centre, Maastricht, Netherlands; Department of Cardiology, Maastricht University Medical Center, Maastricht, the Netherlands; Cardiovascular Research Institute Maastricht, Maastricht, Netherlands; Department of Cardiology, Maastricht University Medical Center, Maastricht, the Netherlands; Cardiovascular Research Institute Maastricht, Maastricht, Netherlands; Department of Cardiothoracic Surgery, Maastricht University Medical Center, Maastricht, the Netherlands; Cardiovascular Research Institute Maastricht, Maastricht, Netherlands; Department of Cardiothoracic Surgery, Maastricht University Medical Center, Maastricht, the Netherlands; Cardiovascular Research Institute Maastricht, Maastricht, Netherlands; Department of Cardiothoracic Surgery, Maastricht University Medical Center, Maastricht, the Netherlands; Cardiovascular Research Institute Maastricht, Maastricht, Netherlands

**Keywords:** Atrial Fibrillation, Hybrid Ablation, Left Atrial Function, Transthoracic Echocardiography, Strain

## Abstract

**OBJECTIVES:**

Thoracoscopic hybrid ablation is an effective and safe rhythm control strategy for patients with complex forms of atrial fibrillation. Its effect on left atrial function has not yet been studied.

**METHODS:**

In a retrospective single-centre analysis of patients undergoing thoracoscopic hybrid ablation, the left atrial emptying fraction was calculated using the biplane modified Simpson method in the apical 2- and 4-chamber views on transthoracic echocardiography. Left atrial strain (reservoir, conduction and contractility) was quantified using dedicated software.

**RESULTS:**

Sixty-seven patients were included (mean age 64 years, long-standing persistent atrial fibrillation in 69%, median atrial fibrillation history duration 64 months). At baseline, left atrial function and contractility were poor. The reservoir and contractile strain improved postprocedure compared to baseline [15 (standard deviation (SD): 8) and 17 (SD: 6); P = 0.013; 3 (SD: 5) and 4 (SD: 4), P = 0.008], whereas the left atrial volume indexed to the body surface area was reduced [51 ml/m^2^ (SD: 14) and 47 ml/m^2^ (SD: 18), *P *=* *0.0024]. In patients with preoperative (long-standing) persistent atrial fibrillation and in patients with rhythm restoration, improvements in the emptying fraction, (reservoir and contractile) strain and the left ventricular ejection fraction were observed, whereas the left atrial volume decreased (*P *<* *0.05).

**CONCLUSIONS:**

In this cohort of patients with severely diseased left atria, improvement in left atrial contractility and in the emptying fraction after thoracoscopic hybrid ablation for atrial fibrillation in patients with persistent atrial fibrillation is mainly due to rhythm restoration. Interestingly, the procedure itself also results in improved left atrial reservoir strain and reversed left atrial remodelling by reducing left atrial volume.

## Graphical Abstract

**Figure:**
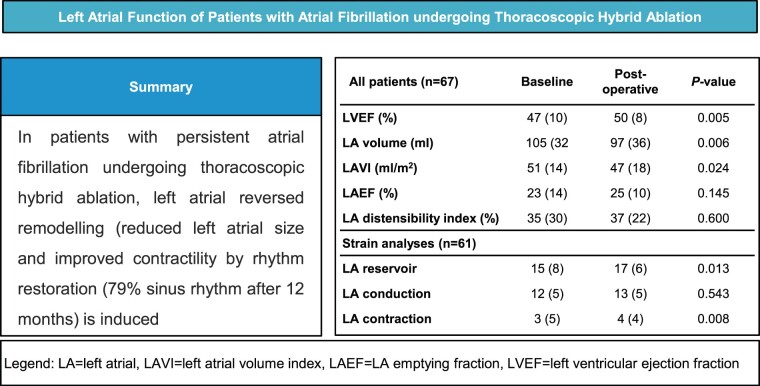

## INTRODUCTION

Recent randomized data have shown that thoracoscopic hybrid atrial fibrillation (AF) ablation yields better rhythm outcomes than catheter ablation, without increasing complication rates [1]. It is somewhat surprising that freedom from atrial tachyarrhythmias (ATA) is used as the main efficacy outcome for rhythm control strategies [2], whereas changes in functional and structural properties of the left atrium (LA) are not systematically evaluated and could be equally important for the symptoms of patients and their burden of AF.

The slow but progressive pathophysiological process underlying AF is characterized by electrical, contractile and structural remodelling [3]. To some extent, reversed LA remodelling can take place after sinus rhythm (SR) restoration following stand-alone catheter ablation [4] or minimally invasive thoracoscopic surgical ablation [5]. However, the degree of reversed LA remodelling after thoracoscopic hybrid ablation and its relation to rhythm restoration have not been studied. Therefore, we analysed changes in LA function, contractility and size and their relation to rhythm restoration following thoracoscopic hybrid AF ablation.

## PATIENTS AND METHODS

### Ethical statement

This study was approved by the institutional review board and ethics committee (METC 2019–1430) of the Maastricht University Medical Centre and analysed anonymously in accordance with review board guidelines. The study complies with the ethical principles of the Helsinki Declaration. Written patient informed consent was waived by the review board due to the retrospective character of the study.

### Study design and population

In this retrospective single-centre cohort study, all patients who underwent thoracoscopic hybrid AF ablation at the Maastricht University Medical Centre, the Netherlands, between January 2010 and January 2022 were screened. All patients who underwent comprehensive transthoracic echocardiography (TTE) at baseline and between 6 and 24 months postoperatively in our centre and with rhythm follow-up for up to 12 months were included. Patients who were in SR during the baseline TTE, but in AF at the postoperative TTE, were excluded from the analysis.

### Hybrid atrial fibrillation ablation procedure

The procedure for a left-sided or bilateral thoracoscopic hybrid approach has been described in detail elsewhere [6, 7]. In brief, thoracoscopic epicardial ablation of the PVs and the box lesion for LA posterior wall isolation was performed either by using a biparietal bipolar radiofrequency (RF) clamp for the PVs (Isolator, AtriCure, Mason, OH, USA) followed by linear ablation of the roof and inferior lines (Coolrail, AtriCure) or by ablation of the PVs and box using a bipolar biparietal RF clamp in 1 continuous lesion (Gemini-S, Medtronic, Minneapolis, MN, USA). Then, the LA appendage (LAA) was excluded. If necessary, the superior caval vein was ablated as well. Figures showing the unilateral and bilateral clamping approaches for the lesion sets have been published elsewhere [8]. Epicardial and/or endocardial electrophysiological validation of the entrance and exit block of the box was performed, and endocardial touch-up of unintentional epicardial conduction gaps was executed if applicable. Additional endocardial ablation was done if necessary, such as a mitral isthmus line in patients with a perimitral flutter or ablation of atrial tachycardia. In the case of typical atrial flutter or right atrial dilatation, a cavotricuspid isthmus ablation was performed.

### Echocardiographic speckle tracking analysis

**Figure 1.**
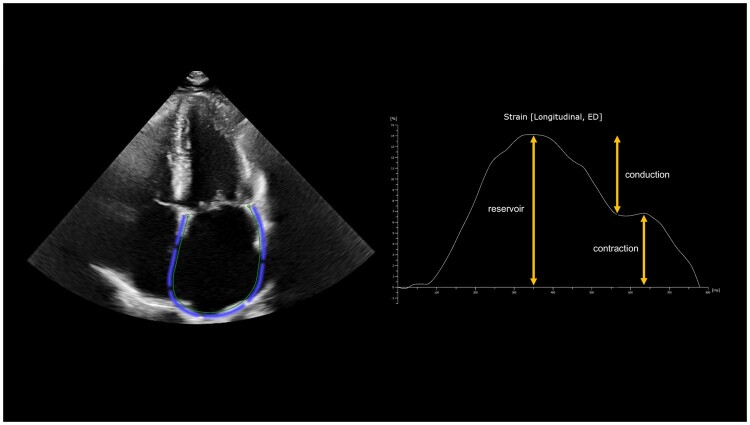
Example of left atrial tracing in the apical 4-chamber view followed by left atrial strain analysis.

Two-dimensional TTEs were performed according to clinical guidelines using a Philips iE33 ultrasound system (Philips Medical Systems, Andover, MA, USA) [9]. All baseline and postoperative measurements took place during routine clinical care by experienced sonographers who were supervised by an experienced imaging cardiologist. Minimal and maximal LA volumes were obtained by manual tracing of the endocardial border of the LA with exclusion of the LAA and the PVs, using the biplane modified Simpson method in the apical 2- and 4-chamber views in end-diastole and end-systole. Subsequently, the LA emptying fraction (LAEF) was calculated [LAEF = (LA_volume_max_–LA_volume_min_)/LA_volume_max_] and averaged from the apical 2- and 4-chamber views. Subsequently, the distensibility index was obtained [(LA_volume_max_–LA_volume_min_)/LA_volume_min_]. Moreover, LA longitudinal strain (reservoir, conduction and contraction) was analysed in a similar fashion using QLAB (version 10, Philips Healthcare, Best, the Netherlands and Andover, MA, USA) (Fig. 1). All strain measurements were performed by a single assessor (C.A.J. van der H.) who was blinded to patient characteristics and outcomes and by a second assessor (B.P.A.) in case of uncertainties. All LAEF measurements were performed by the second assessor (B.P.A.), who was at all times blinded to patient characteristics and outcomes.

### Outcomes and follow-up

The primary outcome was defined as the change in LAEF, LA distensibility index, LA strain (reservoir, conduit and contractile function) and LA volume compared to baseline. The secondary outcome included the change in the left ventricular ejection fraction (LVEF). The association between baseline LA function, contractility and rhythm outcome (freedom from any ATA ≥30 s up to 12 months of follow‐up after a 3-month blanking period) was investigated as well. Because reversed LA remodelling only occurs when SR is maintained, we allowed antiarrhythmic drug (AAD) use for defining rhythm outcome. According to our standard of care, all patients were encouraged to visit the outpatient clinic at 3 and 12 months. Rhythm follow‐up consisted of 24-h, 48‐h or 7‐day Holter monitoring, readout of cardiac implantable electronic devices or 12‐lead ECG monitoring. In case of an ATA recurrence, patient‐tailored treatment conformed to routine care guidelines. All patients were encouraged to undergo a routine TTE examination 12 months postprocedure, although the timing varied for logistical reasons, medical indications or preference of the patient or the physician.

### Statistical analyses

Continuous variables were expressed as mean (SD) or median [1^st^ and 3^rd^ quartile], depending on data distribution. Categorical variables were reported as absolute number and percentage. Subgroups were made based on preoperative AF type [paroxysmal and (long-standing) persistent AF], rhythm outcome after 12 months and rhythm during the baseline and postoperative TTE (SR-SR or AF-SR). Differences in continuous variables between groups were assessed using the independent samples Student *t*-test or the Mann–Whitney *U* test, whereas differences in binary variables were assessed using Pearson’s χ^2^ test or the Fisher exact test. To compare continuous postoperative TTE measurements with baseline values, the paired-samples *t*-test was used. To determine whether postoperative TTE values (overall and between groups based on preoperative AF type) were dependent on the timing of the postoperative TTE, a multivariable linear regression analysis was performed. For each postoperative TTE value, the model was corrected for its baseline value as an important prognostic factor and potential confounder. Thereafter, multivariable binary regression analysis was used to determine whether baseline TTE values could be identified as predictors for rhythm success, corrected for preoperative AF type. All data were analysed using SPSS version 28.0 using a two-sided α of 5% (SPSS Inc., Chicago, IL, USA).

## RESULTS

### Study sample

**Figure 2.**
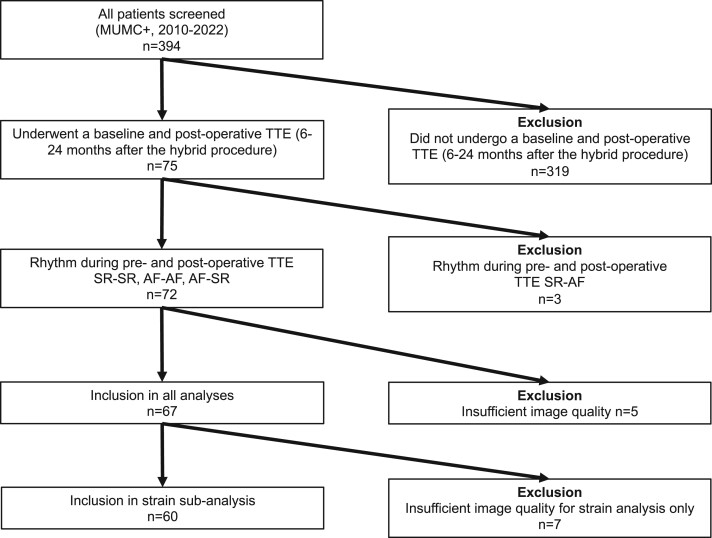
Flow chart of patient inclusion. AF: atrial fibrillation; MUMC+: Maastricht University Medical Centre; SR: sinus rhythm; TTE: transthoracic echocardiography.

Of all patients screened in our centre (*n* = 394), 75 patients received a pre- and a postoperative TTE between 6 and 24 months after the initial hybrid ablation procedure. Of these patients, 3 were in SR during the baseline TTE, but in AF during the postoperative TTE and were therefore excluded from the analysis. Of the remaining patients, 5 had insufficient image quality for speckle tracing analysis and were therefore excluded as well (Fig. 2).

In total, 67 patients were analysed. Baseline characteristics are presented in Table 1. Patients had a mean age of 64 (SD: 8) years; 21% (*n* = 14) were female; the mean BMI was 27 kg/m^2^ (SD: 4) with a median CHA_2_DS_2_-VASc score of 2 [1–3] and a median AF history duration of 64 months [26–129] (Table 1). Moreover, 31% (*n* = 21) had paroxysmal AF (pAF), whereas 69% (*n* = 46) had (long-standing) persistent AF (persAF). Nearly half of all patients had undergone 1 or more previous catheter ablation procedures (*n* = 33, 49%). Moreover, at baseline, 37 out of 67 patients (54%) were taking AAD class I/III prior to their ablation.

**Table 1.** Baseline characteristics and comorbidities of the total population and of subgroups based on preoperative type of atrial fibrillation.

Patient characteristics	All patients n = 67	Preoperative AF type		*P*-value
		pAF, n = 21	persAF, n = 46	
Age (years)	64 (8)	63 (11)	65 (7)	0.516
BMI (kg/m^2^)	27 (4)	27 (4)	28 (4)	0.549
Female (%)	14 (21)	8 (38)	6 (13)	0.027
CHA_2_DS_2_-VASc score	2 [1–3]	2 [1–4]	2 [1–3]	0.674
OSAS (%)	12 (18)	2 (10)	10 (22)	0.314
AF characteristics				
pAF (n, %)	21 (31)	–	–	–
(Long-standing) persAF (n, %)	46 (69)	–	–	–
AF duration (months, IQR)	64 [26–129]	63 [30–136]	64 [20–131]	0.782
Other ATA (n, %)	19 (28)	9 (43)	10 (22)	0.088
Previous CA ablation (n, %))	33 (49)	16 (76)	17 (36)	0.004
TTE				
LVEF (%)	47 (10)	50 (9)	46 (10)	0.070
LA volume (ml)	105 (32)	91 (28)	112 (32)	0.012
LAVI (ml/m^2^)	51 (14)	46 (13)	53 (15)	0.064
LAEF (%)	23 (14)	29 (15)	20 (13)	0.017
LA distensibility index (%)	35 (30)	48 (34)	29 (26)	0.017
Strain				
LA reservoir	15 (8)	18 (6)	13 (8)	0.027
LA conduction	12 (5)	12 (5)	12 (5)	0.884
LA contraction	3 (5)	5 (5)	1 (5)	0.005

AF: atrial fibrillation; ATA: atrial tachyarrhythmia; BMI: body mass index; CA: catheter ablation; CHA_2_DS_2_-VASc: congestive heart failure, hypertension, age ≥75 (doubled), diabetes, stroke (doubled), vascular disease, age 65 to 74 and sex category (female); IQR: interquartile range; LA: left atrium; LAEF: left atrial ejection fraction; LAVI: left atrial volume index; LVEF: left ventricular ejection fraction; OSAS: obstructive sleep apnea syndrome; pAF: paroxysmal AF; persAF: persistent AF; TTE: transthoracic echocardiography.

All patients received 12 months of rhythm monitoring. The median time until postoperative TTE was 12 months (interquartile range 10–15). Rhythm monitoring during follow‐up until 12 months comprised 24-h (*n* = 15, 22%), 48‐h (*n* = 5, 7%), or 7‐day Holter (*n* = 27, 40%) monitoring, readout of cardiac implantable electronic devices (*n* = 4, 6%) or 12‐lead ECG monitoring (*n* = 16, 24%). Of those 16 patients, 8 patients (12%) had ECG monitoring because they already had a recurrence at 6 months and therefore no longer received additional Holter monitoring after 12 months. Furthermore, 2 of these patients (3%) had an ECG only after 12 months because they had 24h Holter monitoring after 24 months instead of after 12 (showing no recurrence) due to the corona virus-19 pandemic. Overall, the rhythm success percentage was 79% allowing AAD (53/67 patients). The 14 failures were treated as follows: electrical cardioversion (ECV) (*n* = 3), redo-catheter ablation (*n* = 3), ECV + redo-catheter ablation (*n* = 2), ECV + AAD (*n* = 1), AAD only (*n* = 1) and no therapy due to the short and asymptomatic character of the recurrence (*n* = 6). Still, patients with recurrences after the 3-month blanking period until the 12-month follow-up, whether or not a rhythm examination was performed, were treated as failures. Based on the available literature and our own experience, normal values of LA function in the healthy population (e.g. patients without a history of AF or other cardiovascular comorbidities) are as follows: LAEF % [30–50], LA reservoir strain [30–40], LA conduit strain [20–30], LA contractile strain [10–20], whereas the normal value of the LA distensibility remains unknown [10, 11]. Not unsurprisingly, baseline LA function and contractility of the current population were low. Slightly better LAEFs and LA distensibility indexes were seen in patients with pAF compared to patients with persAF [29% (SD: 15) vs 20% (SD: 13), *P *=* *0.017 and 48% (SD: 34) vs 29% (SD: 26, *P *=* *0.017, respectively)] (Table 1) and in patients with a successful rhythm outcome at 12 months (*n* = 53, 79%) compared to those with an ATA recurrence (*n* = 14, 21%) [25% (SD: 14) vs 15% (SD: 12), *P *=* *0.029 and 39% (SD: 31) vs 21% (SD: 23), *P *=* *0.029, respectively] (Table S1). Besides, patients with pAF also showed a slightly greater LA reservoir strain [18 (SD: 6) vs 13 (SD: 8), *P *=* *0.027] compared to patients with persAF, whereas LA volume was smaller [91 ml (SD: 28) vs 112 ml (SD: 32), *P *=* *0.013]. However, when the LA volume was normalized for BSA [left atrial voume index (LAVI)], this change was statistically non-significant [46 ml/m^2^ (SD: 13) vs 53 ml/m^2^ (SD: 15) *P *=* *0.064].

### Procedural data

All patients underwent thoracoscopic hybrid ablation for AF consisting of PV isolation and completion of the box lesion. The LAA was excluded in all but 7 patients (10%) due to a low CHA_2_DS_2_-VASc score or safety reasons. In 21 patients (31%), additional epicardial ablation of the superior caval vein was performed. Endocardial touch-up ablation of epicardial conduction gaps was necessary in 14 patients (21%). In the total population, additional endocardial ablation of a cavotricuspid isthmus line was performed in 33 patients (49%), a mitral isthmus line in 4 patients (6%) and ablation of complex fractionated atrial electrograms in 8 patients (12%). No electrophysiological validation was performed in 3 patients (4%) for logistical and/or safety reasons.

### Left atrial function and strain analyses

Overall, the LVEF improved, and both LA volume and the LAVI decreased postprocedure compared to baseline [47% (SD: 10) vs 50% (SD: 8); *P *=* *0.005; 105 ml (SD: 32) vs 97 ml (SD: 36); *P *=* *0.006; and 51 ml/m^2^ (SD: 14) vs 47 ml/m^2^ (SD: 18); *P *=* *0.024, respectively] (Table 2). When stratifying on preoperative AF type, the improvements in LVEF and LA volume were only demonstrated for patients with persAF and not for pAF. In addition, for patients with persAF, improvements in LAEF and the LA distensibility index were seen compared to baseline [20% (SD: 13) vs 24% (SD: 11)1, *P *=* *0.006 and 29 (SD: 26) vs 36 (SD: 23), *P *=* *0.034, respectively] as well as an improvement in the LA reservoir and contractile strain [13 (SD: 8) vs 16 (SD: 6); *P *=* *0.024 and 1 (SD: 5) vs 4 (SD: 4) *P *=* *0.003)].

**Table 2.** Changes in transthoracic echocardiography characteristics following thoracoscopic hybrid atrial fibrillation ablation.

TTE characteristics	Baseline	Postoperative	*P*-value
All patients (n = 67)			
LVEF (%)	47 (10)	50 (8)	0.005
LA volume (ml)	105 (32	97 (36)	0.006
LAVI (ml/m^2^)	51 (14)	47 (18)	0.024
LAEF (%)	23 (14)	25 (10)	0.145
LA distensibility index (%)	35 (30)	37 (22)	0.600
Strain analyses (n = 61)			
LA reservoir	15 (8)	17 (6)	0.013
LA conduction	12 (5)	13 (5)	0.543
LA contraction	3 (5)	4 (4)	0.008
Paroxysmal AF (n = 21)			
LVEF (%)	50 (9)	52 (7)	0.483
LA volume (ml)	91 (28)	88 (42)	0.607
LAVI (ml/m^2^)	46 (13)	43 (20)	0.356
LAEF (%)	29 (15)	26 (8)	0.409
LA distensibility index (%)	48 (34)	38 (18)	0.202
Strain analyses (n = 17)			
LA reservoir	18 (6)	20 (7)	0.322
LA conduction	12 (5)	14 (6)	0.139
LA contraction	5 (5)	6 (4)	0.868
(Long-standing) persAF (n = 46)			
LVEF (%)	46 (10)	50 (9)	0.003
LA volume (ml)	112 (32)	101 (32)	0.003
LAVI (ml/m^2^)	53 (15)	49 (17)	0.039
LAEF (%)	20 (13)	24 (11)	0.006
LA distensibility index (%)	29 (26)	36 (23)	0.034
Strain analyses (n = 43)			
LA reservoir	13 (8)	16 (6)	0.024
LA conduction	12 (5)	12 (5)	0.933
LA contraction	1 (5)	4 (4)	0.003
SR-SR (n = 27)			
LVEF (%)	52 (5)	53 (4)	0.440
LA volume (ml)	98 (33)	96 (38)	0.627
LAVI (ml/m^2^)	47 (14)	46 (17)	0.585
LAEF (%)	35 (13)	30 (11)	0.067
LA distensibility index (%)	61 (32)	47 (24)	0.043
Strain analyses (n = 23)			
LA reservoir	21 (8)	20 (6)	0.343
LA conduction	15 (5)	14 (4)	0.611
LA contraction	7 (6)	6 (4)	0.282
AF-SR (n = 37)			
LVEF (%)	44 (11)	50 (9)	0.004
LA volume (ml)	109 (31)	94 (35)	0.001
LAVI (ml/m^2^)	53 (14)	46 (18)	0.005
LAEF (%)	15 (7)	22 (9)	0.001
LA distensibility index (%)	18 (11)	31 (17)	0.001
Strain analyses (n = 34)			
LA reservoir	10 (4)	16 (4)	0.001
LA conduction	11 (4)	13 (5)	0.206
LA contraction	0 (0)	4 (4)	0.001

Subgroups were made based on preoperative atriofibrillation type and rhythm during the baseline and postoperative transthoracic echocardiography.

AF: atrial fibrillation; LA: left atrial; LAEF: LA emptying fraction; LAV: LA volume; LAVI: LAV-index; LVEF: left ventricular ejection fraction; persAF: persistent AF; SR: sinus rhythm; TTE: transthoracic echocardiography.

Furthermore, although no improvement was seen in LA volume, LA function, LA contractility and LVEF in patients who were in SR during both the pre- and postoperative TTE (SR-SR), these values did improve in patients who were in AF during the baseline TTE and in SR during the postoperative TTE (AF-SR) (Table 2). Overall, none of the baseline TTE parameters were identified as independent predictors for rhythm success at 12 months (Table 3). Finally, an overview of the timing of the postoperative TTE [median 12 (10–15) months] for each patient was given in Supplementary Fig. 1. In patients with persAF and AF-SR, only LA conduction strain was related to the timing of the postoperative TTE [regression coefficient (β) per month = 0.4, 95% confidence interval (0.1, 0.7), *P *=* *0.023 and 0.4, 95% confidence interval (0.1, 0.8), *P *=* *0.014, respectively]. No clinically and statistically significant associations with the timing of the postoperative TTE and the other postoperative TTE values were found (Table S2).

**Table 3.** Overview of the association between baseline transthoracic echocardiography values and rhythm outcome.

TTE characteristics	Success at 12 months (no ATA recurrence)					
	Overall		SR-SR		AF-SR	
	OR [95% CI]	*P*-value	OR [95% CI]	*P*-value	OR [95% CI]	*P*-value
LVEF (%)	1.021 [0.952,1.096]	0.562	0.614 [0.171,2.204]	0.454	1.021 [0.952,1.096]	0.562
LA volume (ml)	0.993 [0.965,1.023]	0.656	1.000 [0.961,1.041]	0.995	0.993 [0.965,1.023]	0.656
LAVI (ml/m^2^)	0.983 [0.923,1.047]	0.591	0.985 [0.906,1.070]	0.714	0.983 [0.923,1.047]	0.591
LAEF (%)	1.109 [0.956,1.286]	0.171	1.052 [0.951,1.164]	0.323	1.109 [0.956,1.286]	0.171
LA distensibility index (%)	1.078 [0.967,1.203]	0.176	1.018 [0.975,1.064]	0.413	1.078 [0.967,1.203]	0.176
LA strain: reservoir	1.042 [0.943,1.153]	0.418	1.003 [0.875,1.149]	0.968	1.210 [0.873,1.676]	0.252
LA strain: conduction	0.984 [0.864,1.121]	0.810	1.022 [0.738,1.416]	0.895	0.940 [0.766,1.153]	0.552
LA strain: contraction	0.944 [0.794,1.122]	0.511	0.998 [0.838,1.189]	0.984	NA	NA

Data are presented as odds ratio followed by the 95% confidence interval.

Subgroups were formed based on rhythm at baseline and postoperative transthoracic echocardiography.

AF: atrial fibrillation; ATA: atrial tachyarrhythmia; LA: left atrium; LAEF: left atrial ejection fraction; LAVI: left atrial volume index; LVEF: left ventricular ejection fraction; OR: odds ratio; SR: sinus rhythm.

## DISCUSSION

This study describes the change in LAEF, LA strain and LA volume based on TTE and the association of baseline values with rhythm outcome 12 months after thoracoscopic hybrid AF ablation. We found that, overall, the procedure leads to improved LA function and contractility and positive LA remodelling. After stratifying based on preoperative type of AF, this finding was only present in patients with persistent AF.

### Left atrial remodelling in atrial fibrillation

Normally, the LA is a contractile compartment that serves as a reservoir, volume sensor and a conduit [12]. In AF, however, 3 types of LA remodelling impacting its function can be distinguished [3, 13]. Initially, electrical remodelling takes place, which is characterized by a shortening of the action potential. Secondly, AF goes hand in hand with functional remodelling and the subsequent loss of LA contractility. This is an important early clinical sign of atrial cardiomyopathy reflecting a reduced LA booster function [14, 15]. The loss of atrial contractility in turn decreases atrial compliance and increases atrial stiffness and dilatation and vice versa [16]. Finally, after prolonged periods of AF, structural remodelling consisting of alterations in atrial geometry and structure is promoted. Because the profound atrial stretch in long-lasting continuous AF can no longer be counteracted by the muscle fibers [17], the synthesis of myocardial and endomysial fibrosis is enhanced and atrial bundles become dissociated [16]. These fibrotic regions with slow conduction velocities increase the stability of AF even further [18], especially when the arrhythmia becomes more complex [19].

Until now, several studies have evaluated reversed LA electrical, contractile and structural remodelling following SR restoration by electrical cardioversion, catheter ablation and minimally invasive surgical ablation. Interestingly, shortening of the atrial refractory period and the impaired rate adaptation are completely reversible within a few days after SR restoration, even after years of AF [18]. However, the occurrence of ATA recurrences after reversed electrical remodelling suggests that the potential for reversed contractile and structural remodelling is a much slower process, sometimes necessitating months to (partially) recover [3].

### Hybrid ablation and left atrial function

The close collaboration between the cardiac surgeon and the electrophysiologist has contributed to good mid-term rhythm outcomes following thoracoscopic hybrid AF ablation [1, 20]. Be that as it may, it remains uncertain whether successful thoracoscopic hybrid AF ablation also results in reversed functional remodelling and a reduction in LA volume. Electrical isolation of the LA posterior wall by definition results in a non-contractile region and may lead to reduced LA contractility. On the other hand, the LA is attached to the body via the PVs, and therefore the posterior wall potentially contributes less to the overall LA contraction than other LA parts. Although epicardial PVI combined with epicardial LA posterior wall isolation is effective in restoring SR in diseased atria with progressed AF [1], substantial healthy atrial myocardial tissue must be preserved in order to retain or even improve atrial function and contractility postprocedure. In that sense, the enthusiasm for big box lesions should be tempered. As long as it is unknown if the size of the box is associated with rhythm outcome, box size should be limited, because isolated tissue does not contract. After all, successful treatment of AF is all about electrical regularization with contractile preservation. It is important to note that, in our study, 90% of all patients underwent LAA exclusion. On the one hand, it is suggested that LAA exclusion contributes to an improved rhythm outcome by isolation of potential triggers arising from the LAA [21] and reduction of the LA critical mass. On the other hand, LA compliance and reservoir function are potentially impaired in the acute phase after LAA exclusion [22]. Still, even if LAA exclusion affects atrial haemodynamics, we found that LA function, including reservoir function and contractility, improved after hybrid ablation. Surprisingly, this improvement was not straightforward in patients with pAF. In part, this situation can be due to the smaller sample size of patients with pAF (*n* = 21, 31%), or potentially because patients with persAF are more likely to be in AF during the baseline TTE. Also, patients with persAF probably had more advanced underlying LA substrate, which potentially allowed for a greater improvement in LA function to be detected over time. In contrast to our results, the meta-analysis by Xiong *et al.* found that the LAEF improved for patients with pAF and not for those with persAF, but after catheter ablation [4]. Because catheter ablation often results in disappointing efficacy outcomes in patients with persAF, independent of the ablation strategy applied [23], it may fail to improve electrical, let alone contractile, remodelling for persAF patients.

### Hybrid ablation and left atrium reversed remodelling

We analysed patients with predominantly persAF with a long median AF history duration of 64 months (25–127), dilated LA and frequent previous catheter ablations that were ablated in a high-expertise tertiary referral hospital. Therefore, not unsurprisingly, the baseline LA strain, the LAEF and the LA distensibility index were low. After the procedure, LA function and contractility remained poor, but improved slightly in patients with persAF and in patients who were in AF during the baseline TTE but in SR during the postoperative TTE. The fact that during AF only the passive LA emptying fraction can be measured and no active contraction is present, it is not surprising that the LAEF and LA contractility strain improved after restoration of rhythm. The improvement in LVEF can consequently be explained by the fact that the atrial kick may improve left ventricular filling, especially in patients with a history of tachycardiomyopathy [24].

As in our study, the improvements in LA function and contractility were observed only in the AF-SR group but not in the SR-SR group. These results stand in contrast to the results of La Meir *et al.* [5]. In their study, an improvement in LA function (LA strain and LAEF) and a decrease in LAVI of at least 15% after successful minimally invasive thoracoscopic ablation was observed in patients who were in SR during both the pre- and postoperative TTE. It must be noted that these patients all had paroxysmal, lone AF, whereas our population consisted mainly of patients with (long-standing) persAF with severely diseased LA and other (cardiovascular) comorbidities, resulting in a severe underlying structural substrate that after years of AF may be (partially) beyond repair. Similarly, we previously reported an improvement in the LAEF measured during SR at baseline and postprocedure after left-sided thoracoscopic ablation combined with a minimally invasive direct bypass grafting procedure [25]. Again, compared to the current study, more patients had pAF (61% vs 31%) and the LAVI was smaller [42 ml/m^2^ (SD: 11) vs 51 ml/m^2^ (SD: 14)].

Interestingly, in the current study, an improvement in LA reservoir strain and a decrease in LA volume were observed as well, which cannot just be attributed to rhythm restoration. Because the measurement of the maximal LA volume is independent of the underlying atrial rhythm, and the LA volume and LA reservoir function can be measured during both SR and AF [26], this implies that the hybrid procedure did result in, at least to some extent, reversed LA remodelling by reducing LA size.

Moreover, in the paper by Voeller *et al.*, all pigs undergoing the Cox-maze procedure as well as the pigs in the control group undergoing only sternotomy and pericardiotomy showed an increase in LA volume and a decrease in overall LA function after 30 days [27]. In our study, we found an overall positive change in overall LA function after a median time of 12 months postprocedure. As such, even if epicardial adhesions caused by the pericardiotomy alone negatively influence LA function in the short term, positive (reversed) LA remodelling is likely to occur in the long term due to rhythm restoration.

## LIMITATIONS

Our results are based on a retrospective single-centre observational study design, including a limited number of patients for subgroup analysis. For example, the lack of improvement in patients with pAF and SR-SR may be due to limited statistical power. Secondly, potential selection bias was present because only patients that underwent a pre- and postoperative TTE of sufficient quality between 6 and 24 months after the thoracoscopic hybrid ablation procedure could be included in the analysis. Thirdly, the use of different monitoring devices to detect ATA recurrence may potentially have led to detection bias. Fourthly, while we looked at the association between postoperative TTE values and the TTE timing, no repeated measure analysis could be performed. Moreover, semi-automatic, speckle tracking imaging of the LA is a sensitive measurement and highly dependent on acute changes in cardiac loading. Furthermore, no comparison in LA function was done between patients who did and did not undergo LAA exclusion because the subgroups were underpowered. Moreover, LAEF and LA contractility were analysed during both AF and SR at baseline and compared with postoperative values in SR. Obviously, rhythm during the TTE is an important factor that inherently influences LA contractility strain and LAEF. Still, in our cohort, comprised of severely diseased patients with complex, long-lasting AF who already had poor LA function and contractility at baseline, it is questionable whether it is even possible to adequately measure baseline LA contractility and LAEF in SR in these patients. Even if all patients were cardioverted to SR prior to the baseline TTE, there is an unknown time period between the moment of the cardioversion and the potential improvement in contractility after SR restoration. In the study by Voeller *et al.*, restoration of SR after the Cox-maze procedure did not immediately result in improved contractility, probably due to a period of atrial stunning [27]. Moreover, in such complex patients, electrical cardioversion alone is often insufficient to restore SR because AF recurs rapidly, making it impossible even to measure contractility and LAEF during SR in those patients. Finally, although previous reports have illustrated that arrhythmia surgery for AF, including hybrid ablation, improves quality of life (QOL)[28], no patient-reported outcomes were measured to determine the relation between the improvement of LA function, rhythm outcome and QOL.

## CONCLUSION

Thoracoscopic hybrid ablation is not only an effective rhythm control strategy (79% freedom from ATA recurrences), but it consequently also results in improved LA function and contractility in patients with persistent atrial fibrillation. Because the improvements in LA function and contractility in this diseased patient cohort seem to be mainly related to SR restoration, (early) rhythm control management is recommended. Moreover, the procedure led to an improvement in the LA reservoir strain and a decrease in LA volume. Because these improvements cannot solely be attributed to rhythm restoration, this result suggests that the hybrid procedure does induce reversed (structural) atrial remodelling. Because the main target of AF therapy is improving AF symptoms, future studies should also study the association between LA functional remodelling after hybrid AF ablation and QOL. Moreover, due to the more extensively present epicardial adhesions following pericardiotomy after a thoracoscopic procedure, but often more successful rhythm outcomes compared to an endocardial catheter ablation procedure, it would also be interesting to compare short- and long-term LA function in ablation-naive patients following thoracoscopy with catheter ablation.

## Supplementary Material

ivae061_Supplementary_Data

## Data Availability

Data are available on reasonable request to the corresponding author.

## ABBREVIATIONS

AF: Atrial fibrillation
AAD: Anti-arrhythmic drug
LA: Left atrium
LAA: LA appendage
LAEF: LA emptying fraction
LAVI: LA volume index
LVEF: Left ventricular ejection fraction
pAF: Paroxysmal AF
persAF: Persistent AF
QOL: Quality of life
SR: Sinus rhythm
TTE: Transthoracic echocardiography
