# Supplementary material for: Left atrial function of patients with atrial fibrillation undergoing thoracoscopic hybrid ablation
Source: Interdiscip Cardiovasc Thorac Surg. 2024 Apr 3;38(4):ivae061. doi: 10.1093/icvts/ivae061 (PMC11043019; doi:10.1093/icvts/ivae061)
Supplement: ivae061_Supplementary_Data [file ivae061_supplementary_data.docx]

**Supplements**

**Tables and figures legends**

**Table S1.** ATA=atrial tachyarrhythmia; AAD=anti-arrhythmic drug; BMI=body mass index; CA=catheter ablation; LA=left atrial; LAV=LA volume; LAVI=LAV-index; LVEF=left ventricular ejection fraction; OSAS=obstructive sleep apnea syndrome; TTE=transthoracic echocardiography.

**Table S2.** β=regression coefficient; LA=left atrial; LAEF=LA emptying fraction; LAV=LA volume; LAVI=LAV-index; LVEF=left ventricular ejection fraction; NA=not applicable; pAF=paroxysmal AF; TTE=transthoracic echocardiography.

**Figure S1.** For each patient (y-axis) the timing of the postoperative TTE as well as the type (symbol and color) and timing (months) of rhythm monitoring was indicated. Square (yellow)=Holter; circle (light blue)=ECG; triangle (green)=post-operative TTE. Detected recurrences are colored in red.

**Table S1.** Baseline characteristics and comorbidities of the total population and of subgroups based on rhythm outcome until 12 months of follow-up.

| Patient characteristics | | All patients  n=67 | Rhythm outcome 12 months | | *P*-value |
| --- | --- | --- | --- | --- | --- |
|  |  |  | SR (n=53) | ATA (n=14) |  |
| Age (years) | | 64 (8) | 64 (9) | 66 (8) | 0.411 |
| BMI (kg/m^2^) | | 27 (4) | 28 (4) | 26 (3) | 0.242 |
| Female (%) | | 14 (21) | 11 (21) | 3 (21) | 1.000 |
| CHA_2_DS_2_-VASc | | 2 [1–3] | 2 [1–3] | 2 [1–4] | 0.529 |
| OSAS (%) | | 12 (18) | 9 (17) | 3 (21) | 0.704 |
| AF characteristics | |  |  |  |  |
|  | pAF (n, %) | 21 (31) | 18 (34) | 3 (21) | 0.522 |
|  | (longstanding)-persAF (n, %) | 46 (69) | 35 (66) | 11 (79) | 0.522 |
|  | AF duration (months, IQR) | 64 [26–129] | 59 [20–122] | 71 [46–150] | 0.301 |
|  | Other ATA (n, %) | 19 (28) | 15 (28) | 4 (29) | 1.000 |
|  | Previous CA ablation (n, %)) | 33 (49) | 26 (49) | 7 (50) | 1.000 |
| TTE | |  |  |  |  |
|  | LVEF (%) | 47 (10) | 48 (10) | 45 (11) | 0.265 |
|  | LA volume (ml) | 105 (32) | 104 (33) | 110 (28) | 0.495 |
|  | LAVI (ml/m^2^) | 51 (14) | 50 (15) | 54 (13) | 0.323 |
|  | LAEF (%) | 23 (14) | 25 (14) | 15 (12) | 0.029 |
|  | LA distensibility index (%) | 35 (30) | 39 (31) | 21 (23) | 0.029 |
|  | **Strain** |  |  |  |  |
|  | LA reservoir | 15 (8) | 15 (8) | 12 (7) | 0.287 |
|  | LA conduction | 12 (5) | 12 (5) | 12 (5) | 0.798 |
|  | LA contraction | 3 (5) | 3 (6) | 1 (3) | 0.162 |

**Table S2.** The association between the timing of the post-operative TTE (months) per post-operative TTE value.

**A.** Subgroups based on pre-operative AF type.

| TTE characteristics | Overall | | pAF | | pers-AF | |
| --- | --- | --- | --- | --- | --- | --- |
|  | β [95% CI] | *P*-value | β [95% CI] | *P*-value | β [95% CI] | *P*-value |
| LVEF (%) | -0.1 [-0.4, 0.3] | 0.753 | -0.1 [-0.8, 0.5] | 0.738 | -0.1 [-0.5, 0.4] | 0.798 |
| LA volume (ml) | -0.7 [-2.0, 0.6] | 0.275 | -1.0 [-3.3, 1.3] | 0.389 | -0.3 [-1.9, 1.3] | 0.706 |
| LAVI (ml/m^2^) | -0.4 [-1.1, 0.3] | 0.252 | -0.8 [-2.0, 0.4] | 0.191 | -0.2 [-1.1, 0.7] | 0.702 |
| LAEF (%) | 0.0 [-0.5, 0.5] | 0.953 | 0.2 [-0.5, 1.0] | 0.520 | -0.3 [-0.9, 0.3] | 0.359 |
| Distensibility (%) | -0.1 [-1.1, 0.9] | 0.836 | 0.6 [-1.1, 2.2] | 0.484 | -0.8 [-2.0, 0.4] | 0.185 |
| LA strain: reservoir | 0.0 [-0.4, 0.3] | 0.868 | 0.1 [-0.7, 0.9] | 0.790 | -0.1 [-0.5, 0.3] | 0.658 |
| LA strain: conduction | 0.3 [0.0, 0.6] | 0.037 | 0.1 [-0.4, 0.7] | 0.589 | 0.4 [0.1, 0.7] | 0.023 |
| LA strain: contraction | 0.2 [0.0, 0.4] | 0.053 | -0.2 [-0.6, 0.3] | 0.463 | -0.2 [-0.5, 0.0] | 0.088 |

**B.** Subgroups based on rhythm during the pre- and post-operative TTE.

| TTE characteristics | SR-SR | | AF-SR | |
| --- | --- | --- | --- | --- |
|  | β [95% CI] | *P*-value | β [95% CI] | *P*-value |
| LVEF (%) | 0.1 [-0.3,0.4] | 0.632 | -0.3 [-0.9,0.2] | 0.251 |
| LA volume (ml) | -1.0 [-3.2,1.2] | 0.365 | 0.2 [-1.4,1.7] | 0.816 |
| LAVI (ml/m^2^) | -0.5 [-1.5,0.6] | 0.365 | 0.0 [-1.0,0.9] | 0.931 |
| LAEF (%) | 0.2 [0.7,1.1] | 0.650 | -0.3 [-0.9,0.3] | 0.325 |
| Distensibility (%) | 0.4 [-1.7,2.5] | 0.691 | -0.7 [-1.9,0.4] | 0.206 |
| LA strain: reservoir | 0.5 [-0.2,1.1] | 0.159 | -0.1 [-0.5,0.3] | 0.601 |
| LA strain: conduction | -0.2 [-0.7,0.2] | 0.307 | 0.4 [0.1,0.8] | 0.014 |
| LA strain: contraction | -0.3 [-0.6,0.1] | 0.205 | NA | NA |

**Figure S1.** Overview of the timing of each postoperative TTE during follow-up.
